# The effect of low dose caffeine powder supplementation on serve speed, spike speed, and speed-endurance in elite sitting volleyball players: a randomized double-blind crossover study

**DOI:** 10.1186/s13102-025-01408-8

**Published:** 2025-11-06

**Authors:** Azize Bingol Diedhiou, Dilara Erkan, Melek Guler, Halit Sar, Izzet Karakulak, Ender Eyuboglu, Mehmet Can Gundem, Raci Karayigit, Selin Yildirim Tuncer, Ulas Can Yildirim

**Affiliations:** 1 Department of Coaching Education, School of Physical Education and Sports, Şırnak University, Central, Şırnak, 73000 Türkiye; 2 Department of Physical Education and Sports, Institute of Health Sciences, Ankara University, Gölbaşı, Ankara 06830 Türkiye; 3 Department of Physical Education and Sports, Faculty of Sport Sciences, Karamanoğlu Mehmetbey University, Central, Karaman, 70200 Türkiye; 4 Department of Physical Education and Sports, Faculty of Sport Sciences, Sinop University, 57100 Osmaniye, Sinop Türkiye; 5 Department of Sport Management, Faculty of Sport Sciences, Mardin Artuklu University, Artuklu, Mardin 47100 Türkiye; 6 Department of Coaching Education, Faculty of Sport Sciences, Bartın University, Central, Bartın, 74100 Türkiye; 7 Department of Sport Management, Faculty of Sport Sciences, Sinop University, Osmaniye, Sinop 57100 Türkiye; 8 Department of Coaching Education, Faculty of Sport Sciences, Ankara University, Gölbaşı, Ankara 06830 Türkiye; 9 Department of Coaching Education, Faculty of Sport Sciences, Lokman Hekim University, Çankaya, Ankara 06510 Türkiye; 10 Department of Coaching Education, Faculty of Sport Sciences, Sinop University, Osmaniye, Sinop 57100 Türkiye

**Keywords:** Caffeine 1, Sitting volleyball 2, Serve speed 3, Spike speed 4, Speed-Endurance 5

## Abstract

**Background:**

Sitting volleyball relies heavily on upper-body strength and anaerobic capacity. Serve, spike, and speed-endurance are decisive skills, yet the ergogenic potential of low-dose caffeine in this Paralympic sport remains unclear.

**Purpose:**

To examine the acute effects of low-dose caffeine (3 mg/kg) supplementation on serve speed, spike speed, and speed-endurance in elite sitting volleyball players.

**Methods:**

Using a randomized, double-blind, crossover design, 13 elite male athletes from the Turkish National Sitting Volleyball Team completed serve speed, spike speed, and speed-endurance tests under caffeine (CAF) and placebo (PLA) conditions.

**Results:**

Caffeine intake produced a moderate improvement in serve speed (*p* = 0.028, d = 0.460); however, this effect did not remain statistically significant after Bonferroni correction (adjusted *p* = 0.084). No significant effects were observed for spike speed (*p* = 0.547, d = 0.166) or speed-endurance performance (*p* = 0.709, d = 0.111). Perceived exertion during the speed-endurance test was similarly high in both conditions.

**Conclusions:**

Low-dose caffeine may offer a trend toward improved serve performance, but the effect was not robust after statistical adjustment, and no benefits were observed for spike speed or speed-endurance. These findings highlight that caffeine’s ergogenic effects are context-dependent and shaped by task complexity and sport-specific motor demands. Further research with larger and more diverse samples, genotype-based subgroups, and varied dosing strategies is warranted to clarify caffeine’s role in adaptive sports.

**Trial registration:**

The randomized controlled trial was retrospectively registered on 21/06/2025 at ClinicalTrials.gov, under the registration number NCT07056231.

## Introduction

Sitting volleyball is a Paralympic sport discipline in which players must possess well-developed upper body physical fitness and trunk muscle strength, alongside other essential physiological attributes that contribute to their overall performance [1]. These physical qualities are crucial as they facilitate the execution of fundamental movements and techniques in sitting volleyball, where lower limb involvement is either limited or entirely absent. The increasing interest in sitting volleyball in the international arena has increased the demand for reliable, precise, and accurate analysis and evaluation of the performance levels of players and teams. Despite the popularity of the Paralympic sport, there is a lack of adequate research on competitive outcomes and the factors that influence them in sitting volleyball. In a study examining the relationship between specific components and game efficiency, service test scores of sitting volleyball athletes showed significant and positive correlations with fundamental game elements such as attack, block and defense efficiency [2]. Another study on sitting volleyball players emphasized that service efficiency can determine the final score [3]. However, the parameters of service speed and match result were not specifically addressed. Given that upper body strength and explosive power play a dominant role in generating high ball velocities, these skills significantly influence the competitive dynamics of the game in sitting volleyball. Specifically, an effective serve, characterized by a high-speed ball trajectory, can disrupt the opposing team’s service reception performance and subsequently affect their attack tempo, thereby conferring a strategic advantage to the serving team [4, 5]. While these findings have been extensively examined in the context of standing volleyball, they strongly suggest that similar principles may apply to sitting volleyball, where upper body attributes are even more critical due to the absence of lower limb contribution. Consequently, serve and spike velocity are likely to play a decisive role in performance outcomes for sitting volleyball players as well. Another fundamental factor influencing match performance in sitting volleyball is speed-endurance capacity. This physical feature determines the players’ capacity to perform high-tempo and high-intensity movements for a long time. Furthermore the relationship between speed-endurance levels and match success has been validated through statistically significant results, indicating its pivotal role in competitive play [6, 7]. Especially in sitting volleyball, a sport where repetitive explosive movements are frequently performed, a high level of speed-endurance plays a critical role in terms of sustainable performance throughout the match. In this context, the importance of serve speed and speed-endurance performance is evident. However, there is a lack of studies. It is thought that ergogenic aids can constitute a practical option for the development of these skills. Publications in the literature report that Paralympic athletes use less ergogenic aids than non-disabled athletes [8–10]. Nonetheless, it is mentioned that the use of supplements by Paralympic athletes increased between 2004 and 2012 [11]. Ergogenic substances, commonly used by non-disabled athletes to enhance athletic performance, caught the attention of the research team conducting the current study because of their potential to maximize serve-spike speed and speed-endurance performance in sitting volleyball players.

Among supplements, caffeine consumption has been extensively studied in the field of sports sciences for its performance-enhancing effects, especially in relation to its effect on alertness, reaction time, and neuromuscular coordination. The main mechanism of caffeine’s performance-enhancing effect is adenosine receptor antagonism. Caffeine primarily causes an increase in neurotransmitter release via adenosine antagonism in the central nervous system (CNS) [12, 13]. The main effect of adenosine, a neuromodulator with inhibitory functions, is to reduce the concentration of various neurotransmitters, including serotonin, dopamine, acetylcholine, norepinephrine, and glutamate. Since caffeine has a molecular structure similar to adenosine, it increases the concentration of these neurotransmitters by binding to adenosine receptors [13–15]. The impact of these physiological mechanisms on sports performance is particularly evident during short-duration, high-intensity efforts. Indeed, a study that may be relevant to sitting volleyball reported that caffeine consumption can improve short-duration, high-intensity upper extremity performance in paraplegic athletes [16]. Furthermore, given the correlation between anaerobic capacity and serve and spike execution efficiency in sitting volleyball [7], it can be suggested that caffeine’s ergogenic properties may be a potential tool for enhancing these key performance elements. In additionally, Marszalek et. all [7] reported that speed-endurance is positively correlated with anaerobic power and upper body strength. Therefore, the effect of caffeine on the upper extremities will indirectly have a positive impact on speed and endurance. Furthermore, scientific studies have shown that caffeine has performance-enhancing effects in non-disabled athletes, especially in speed-endurance races (activities lasting 60–180 s) and repetitive sprint races. A study conducted by Bell and McLellan [17] provides findings supporting the positive effects of caffeine consumption on such exercises. Overall, it is thought that caffeine use has the potential to increase athletic performance in sitting volleyball.

Previous studies have shown that caffeine consumption produces different physiological and performance effects depending on the dose. Low and moderate doses (3–6 mg/kg) of caffeine have been reported to provide positive ergogenic effects on athletic performance, while high doses (9 mg/kg and above) have been reported to cause adverse physiological reactions such as irritability, insomnia, stomach upset, and, in some cases, cardiac tachycardia [18]. To achieve optimal performance gains, it is crucial to increase scientific studies supporting the ergogenic benefits of caffeine use, especially at low doses. Based on prior evidence, a 3 mg/kg dose was selected to balance performance benefits with a reduced risk of side effects.

Despite the growing interest in sitting volleyball as a competitive Paralympic sport, a notable gap remains in the literature regarding the effects of ergogenic aids on performance outcomes specific to this discipline [8, 10, 16]. Moreover, the potential of caffeine to enhance critical performance parameters such as serve and spike velocity and speed-endurance has been largely overlooked in this context. Given the decisive role these attributes play in match outcomes, investigating the ergogenic effects of caffeine presents a valuable opportunity to inform evidence-based performance strategies in sitting volleyball. Therefore, the present study aims to examine the acute effects of a low caffeine dose (3 mg/kg) on serve and spike velocity, as well as speed-endurance performance, in sitting volleyball players. By addressing this research gap, the study seeks to contribute novel insights to the adapted sports literature and offer practical guidance to Paralympic athletes and coaches regarding the informed use of caffeine for performance enhancement.

## Materials and methods

### Sample size estimation

The required sample size was estimated a priori using G*Power software (version 3.1.9.7, Düsseldorf, Germany) for a paired-samples t-test (two-tailed, α = 0.05, 1–β = 0.80). Based on large effect sizes (Cohen’s d ≈ 0.8) previously reported in acute caffeine studies on volleyball players and caffeine outcomes in athletes, the analysis indicated that a minimum of 12 participants would be required to achieve adequate statistical power [19]. In the present study, 13 elite national team sitting volleyball players were recruited, which met this requirement. If a medium effect size (d ≈ 0.5) were assumed, a larger sample would have been necessary. However, due to the unique constraints of working with elite national team athletes in a Paralympic discipline, the sample size was limited to 13.

### Participants

Thirteen elite male sitting volleyball players (29.84 ± 11.50 years, 91.76 ± 16.84 kg weight, 182.61 ± 10,40 cm height, VS1 disable level, 328.2 ± 272.2 mg/day habitual caffeine intake) from the 2024 Paravolley European Champion Turkish Sitting Volleyball Men’s National Team participated in this study. The inclusion criteria were: (1) having a minimum level of disability according to the World ParaVolley classification, (2) not having any neuromuscular or musculoskeletal disorders that would prevent participation in the measurements, (3) not smoking or consuming alcohol, and (4) not using any ergogenic support to increase performance in the last 3 months. Exclusion criteria were: (1) failure to complete all test sessions, (2) occurrence of injury or illness during the study period that could affect performance and (3) changes in habitual caffeine consumption during the study.

**Fig. 1:**
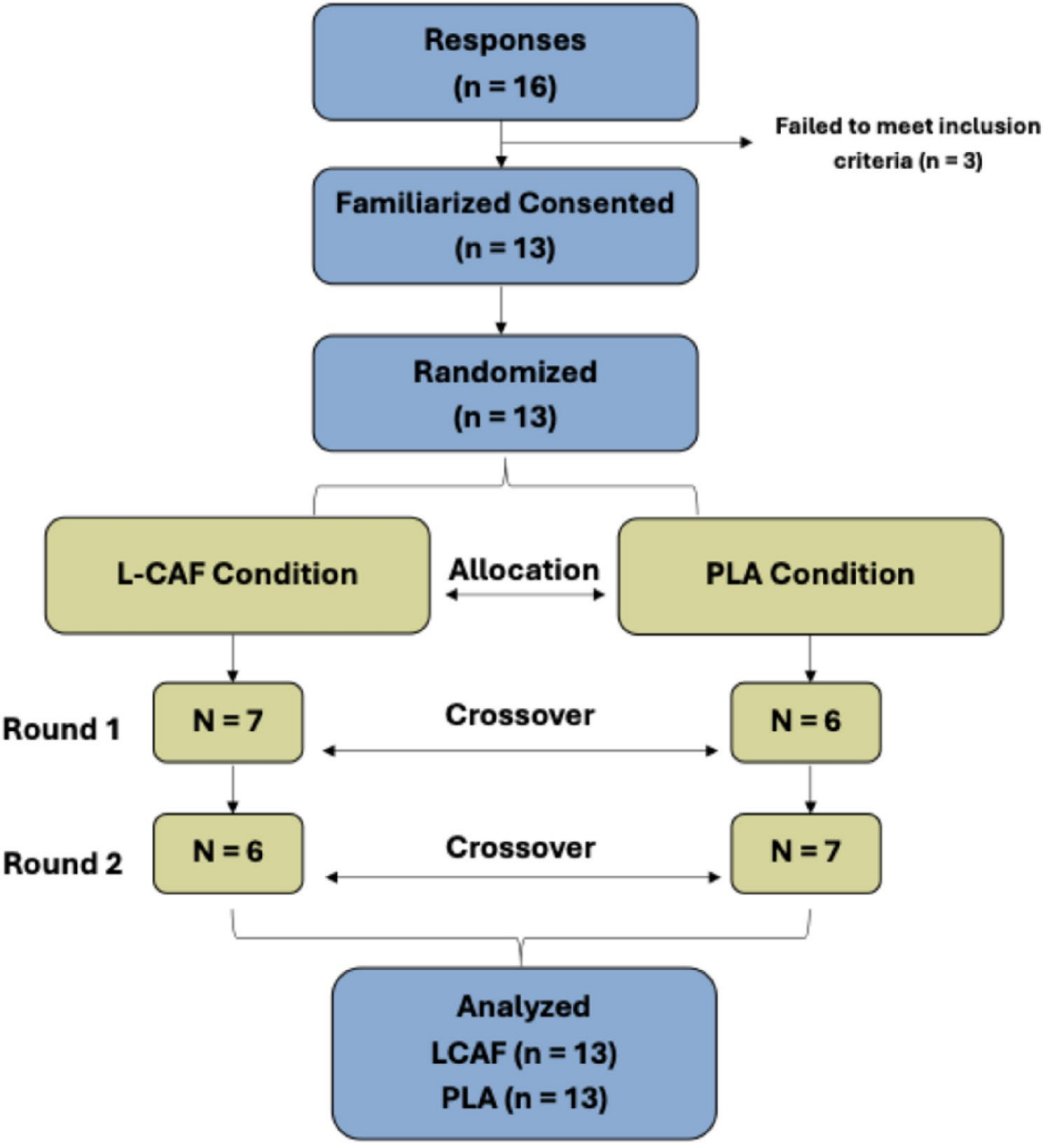
CONSORT flowchart of the study

All athletes were given detailed information about the study, their voluntary participation was ensured, and an informed consent form was obtained before any measurement. The study was approved by the Sinop University Human Research Ethics Committee (approval no: 2025/91) and was conducted in accordance with the ethical standards of the Declaration of Helsinki [20]. This study adheres to the Consolidated Standards of Reporting Trials (CONSORT) guidelines to ensure transparency and comprehensive reporting of the randomized controlled trial design and methodology (Fig. 1).

### Study design

This study had a randomized, double-blind, counterbalanced, and cross-over design. In this double-blind design, both participants and assessors who recorded the performance data were blinded to the experimental conditions. Athletes participated in a total of three sessions (one familiarization session and two test sessions). Participants were randomized using a computer-based random number generator to receive the two conditions (L-CAF and PLA) in different orders. As required by the crossover design, each participant completed both conditions, with the order of supplementation randomized. A 48-hour washout period was given between each test session, consistent with previous caffeine supplementation studies [21]. All measurements were performed at the same time interval (e.g., 09:00–10:00) in a fasted state (after an overnight fast of approximately 8–10 h) to minimize the possible effects of circadian rhythm differences. Participants were asked not to change their eating habits, to avoid intense exercise, and to consume alcohol during the study. They were advised to maintain their habitual daily caffeine consumption in order to prevent withdrawal symptoms and avoid potential performance decrements that could occur if their usual intake was restricted before testing [5]. Moreover, a validated daily caffeine consumption questionnaire [22] was administered to all participants prior to the testing sessions.

**Fig. 2:**
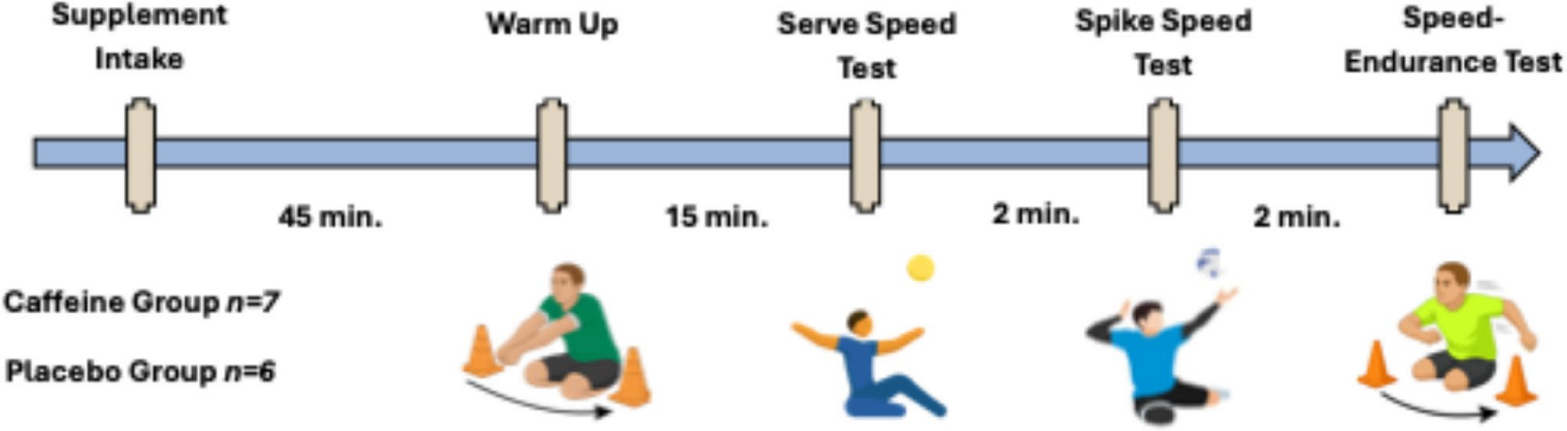
Timeline of daily experimental procedures

In each test session, participants consumed a randomly assigned supplement containing caffeine (LCAF) or placebo (PLA) 45 min before the tests. All participants completed a standard warm-up protocol which consisted of a 5-minute low-intensity general warm-up, followed by 10 min of dynamic stretching and technical work, 15 min before the test. The warm-up protocol was standardized and applied by the same researcher across all sessions to ensure consistency. After the warm-up period, athletes performed the serve speed, spike speed, and speed-endurance performance tests, respectively. For serve and spike tests, the best of three trials was recorded, as a single poor attempt could lower the mean and reduce the reliability of the results. Participants were given a 30-second rest period between trials and a 2-minute rest period between tests (Fig. 2.). These intervals were selected to allow adequate recovery while minimizing testing time and are consistent with previous studies on explosive performance protocols [5, 23]. Rating of Perceived Exertion (RPE) during the speed-endurance course were recorded using the Borg 6–20 scale immediately at the end of the test. All tests were conducted in an indoor sports hall on a suitable for international games surface, under lighting. No external spectators or distractions were present, and only the research team and participants were allowed in the hall to ensure a controlled and standardized testing environment. Caffeine and placebo doses were prepared using an analytical laboratory balance (Shimadzu, Tokyo, Japan) capable of measuring to within precision of 1 mg, and Oxford brand pure caffeine powder (ISO 14001 − 98.5% purity; The Oxford Vitality Health Company ltd; London, UK) and polydextrose (Litesse^®^ Ultra, Danisco USA Inc., Terre Haute, IN, USA) was used throughout the study. Both supplements were provided in powder form and dissolved in 250 mL of water. The solutions were visually identical (transparent), and a non-caloric sweetener was added to mask any potential taste differences. Supplement preparation and administration were conducted in accordance with a double-blind protocol, ensuring that neither the participants nor the investigators conducting the tests were aware of the condition assignments.

### Data collection tools

#### Serve speed

The serve speed of sitting volleyball players was evaluated with a standardized measurement protocol within the framework of World ParaVolley game rules and court dimensions. Participants positioned themselves on the service line with their hips touching the court surface and used a serve. During the test, participants were instructed to hit the ball within the target area at the highest possible speed appropriate to their individual techniques. Each participant was given three attempts, and the best score was recorded. Serve speed measurements were performed with a high-precision handheld radar (Bushnell Velocity Speed Gun, USA). This device has been shown to provide valid and highly reliable measurements of ball speed, with excellent agreement compared to a gold-standard radar (ICC >0.98, typical error ≈ 0.2–0.4 km/h) [24]. During testing, the radar was positioned in line with the ball trajectory according to manufacturer recommendations to ensure consistent sensor placement. All attempts were applied by the same researcher to ensure consistency of execution. The radar device was always positioned at the same height and distance by the same operator.

#### Spike speed

The spike performance was performed by positioning the participants in front of the net in accordance with the real conditions in the game. The participants were instructed to hit the ball at the highest speed possible into the 3 × 3 m target area. Spike speed was measured with the same handheld radar device used for serve speed assessments (Bushnell Velocity Speed Gun, USA). As previously noted, this instrument provides valid and highly reliable measurements of ball speed (ICC >0.98, typical error ≈ 0.2–0.4 km/h) [24]. For spike testing, the radar was positioned in line with the ball trajectory following the manufacturer’s recommendations to ensure consistent placement. For spike testing, the radar was consistently positioned at the same height and distance in line with the ball trajectory, and all attempts were applied by the same researcher to ensure standardization and minimize variability.

#### Speed-endurance performance

**Fig. 3:**
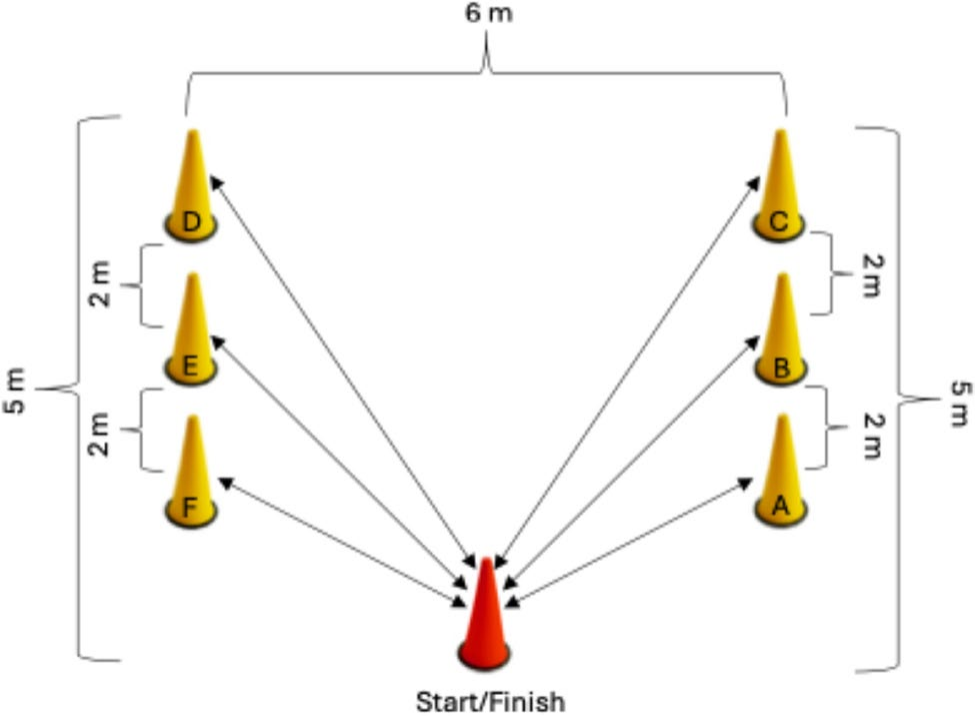
Speed-endurance test parkour

The speed and endurance test developed by Marszalek et al. (Fig. 3) was applied with a single-trial protocol to evaluate the anaerobic endurance capacity of the athletes [7]. The test begins with athletes in a seated position behind the starting point at cone A. The participants completed the specified route by moving back and forth between cones A, B, C, D, E and G respectively in the shortest time possible in accordance with the requirements of the test. All athletes were required to maintain physical contact with the base of each cone throughout the test. All sessions were applied by the same researcher, and verbal cues were given that participants throughout the test. The results were measured using the Optojump photocell system (Microgate, Bolzano, Italy), which records with a resolution of 1/1000 s and has been shown to provide highly valid and reliable measurements [25]. The system was positioned on tripods according to the manufacturer’s recommendations, ensuring consistent alignment and sensor placement throughout testing.

#### Data analysis

All data are reported as mean ± standard deviation (Mean ± SD). Normality of distribution of data was assessed by Shapiro-Wilk normality test. Paired t-test was applied to examine the differences between LCAF and PLA conditions in serve speed, spike speed and speed-endurance performance. Given the presence of multiple comparisons across three outcome variables, Bonferroni correction was applied to control the family-wise error rate. The adjusted significance threshold was set at α = 0.0167 (0.05/3). In addition, 95% confidence intervals (CIs) were calculated and reported for mean differences and effect sizes (Cohen’s d) to provide estimates of precision. To further assess potential order or carryover effects inherent in the crossover design, difference scores (L-CAF – PLA) were computed for each outcome, and independent-samples t-tests were conducted to compare participants who started with the L-CAF condition versus those who started with the PLA condition.

Power analysis was conducted using G*Power for a paired-samples t-test (two-tailed, α = 0.05, *n* = 13, power = 0.80). Cohen’s d coefficient was calculated for effect sizes based on the mean difference divided by the standard deviation of the paired differences, thereby accounting for within-subject variability in the crossover design. Effect sizes were interpreted according to the following classification: insignificant (< 0.2), small (0.2–0.5), medium (0.5–0.8), and large (>0.8) [26]. All analyses were performed using IBM SPSS Statistics 25.0 (IBM Corp., Armonk, NY, USA) software.

## Results

The following section summarizes the outcomes of the crossover trial, comparing performance and perceptual measures between the L-CAF and PLA conditions, with results reported as means ± SD, confidence intervals, and effect sizes.

**Fig. 4:**
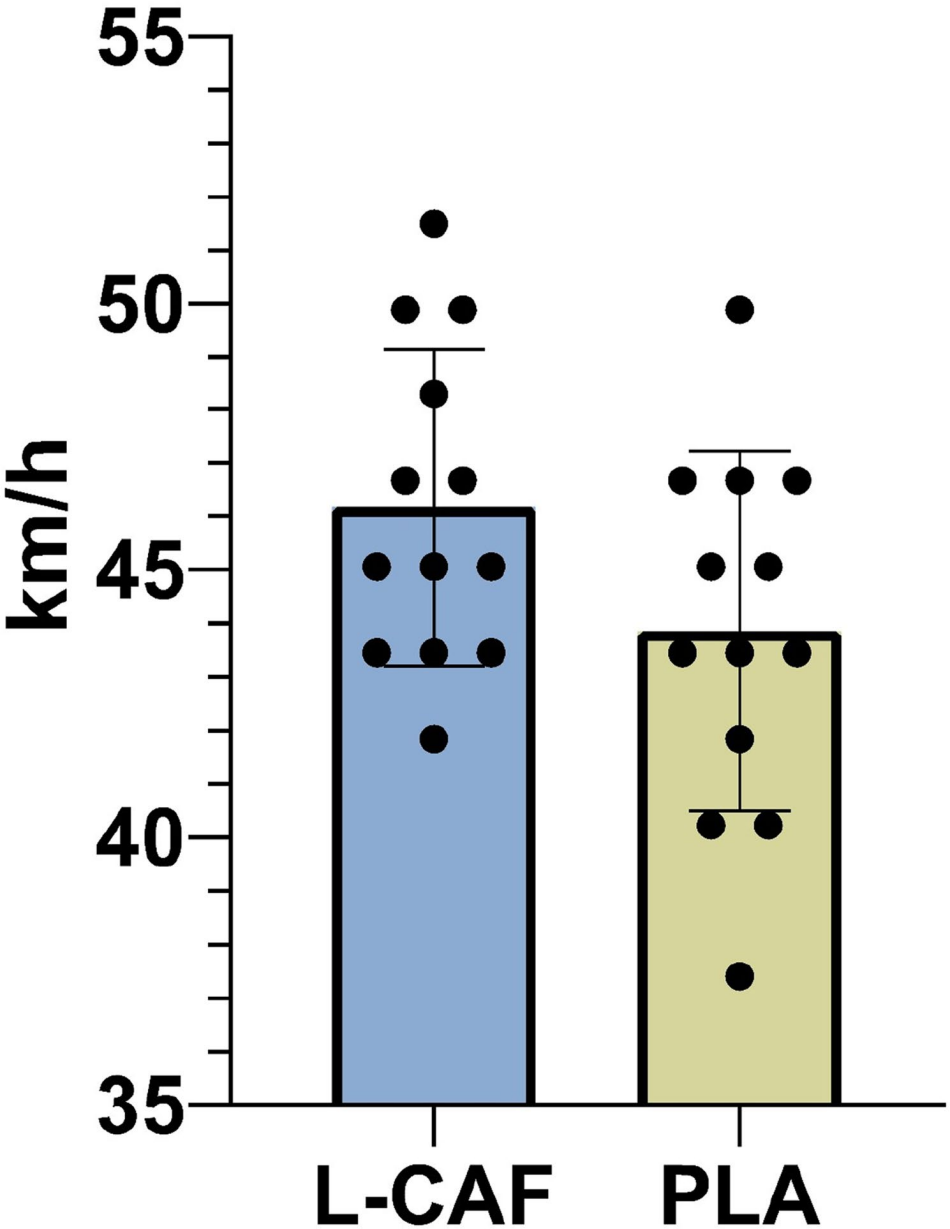
Effects of low-dose caffeine on serve speed performance compared to placebo

A significant difference was found between the L-CAF and PLA conditions in serve speed (t = 2.501, *p* = 0.028), with the L-CAF condition showed significantly higher serve speeds (45.30 ± 5.74 km/h ‘28.15 ± 3.57 mi/h’) than the PLA condition (42.69 ± 5.52 km/h ‘26.53 ± 3.43 mi/h’) (Fig. 4.). The mean difference was 1.62 km/h (95% CI: 0.21 to 3.02), with a medium effect size (Cohen’s d = 0.460, 95% CI: − 0.175 to 1.095). However, after applying the Bonferroni correction to account for multiple comparisons across the three primary outcomes (serve, spike, and speed-endurance), the adjusted significance threshold was set at α = 0.0167. Under this more stringent criterion, the difference in serve speed was no longer statistically significant (adjusted *p* = 0.084) (Table 1.).

**Table 1:** Comparison of serve speed, spike speed, and speed-endurance performance between low-dose caffeine and placebo

PARAMETERS		M ± SD	t	*p*	p_adj	95% CI	d	d (95% CI)
Serve Speed *a=*(mi/h); *b=* (km/h)	*L-CAF n = 13*	*a =* 28.15 ± 3.57 *b =* 45.30 ± 5.74	2.501	0.028*	0.084	1.62	0.460	−0.175–1.095
	*PLA n = 13*	*a =* 26.53 ± 3.43 *b =* 42.69 ± 5.52						
Spike Speed *a=* (mi/h); *b=* (km/h)	*L-CAF n = 13*	*a =* 28.92 ± 6.55 *b =* 46.54 ± 10.54	0.619	0.547	1.00	1.23	0.166	−0.442–0.774
	*PLA n = 13*	*a =* 27.69 ± 8.16 *b =* 44.56 ± 13.13						
Speed-Endurance (seconds)	*L-CAF n = 13*	24.38 ± 3.66	0.382	0.709	1.00	0.40	0.111	−0.495–0.717
	*PLA n = 13*	23.98 ± 3.57						

* *p* < 0,05

**Fig. 5:**
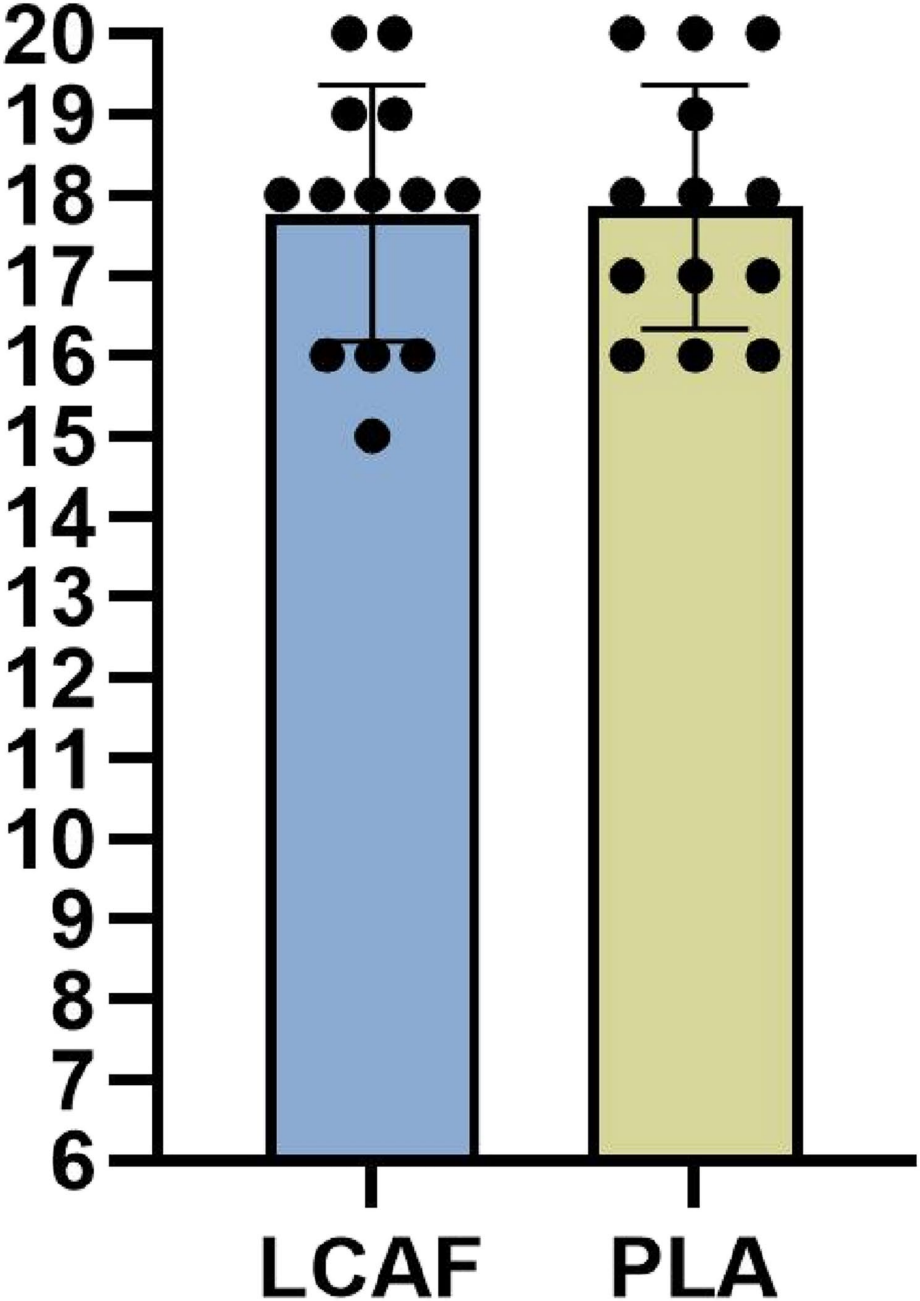
Post-speed-endurance test RPE scores following L-CAF and PLA conditions

On the other hand, no significant difference was found between the L-CAF and PLA conditions in spike speed (t = 0.619, *p* = 0.547) and speed-endurance (t = 0.382, *p* = 0.709) variables. For spike speed, the L-CAF condition (46.54 ± 10.54 km/h [28.92 ± 6.55 mi/h]) showed a slightly higher mean compared to the PLA condition (44.56 ± 13.13 km/h [27.69 ± 8.16 mi/h]), with a mean difference of 1.23 km/h (95% CI: − 3.10 to 5.56) and a small effect size (Cohen’s d = 0.166, 95% CI: − 0.442 to 0.774). Similarly, the speed-endurance performance showed a small mean difference of 0.40 s (95% CI: − 1.90 to 2.71) between the L-CAF (24.38 ± 3.66 s) and PLA (23.98 ± 3.57 s) conditions, with a trivial effect size (Cohen’s d = 0.111, 95% CI: − 0.495 to 0.717) (Table 1). Additionally, post-speed-endurance test RPE values were 17.76 ± 1.58 in the L-CAF condition and 17.84 ± 1.51 in the PLA condition. The difference between conditions was not statistically significant (t= −0.113, *p* = 0.912) (Fig. 5).

To further assess potential order effects, difference scores (L-CAF – PLA) were compared between participants who started with the L-CAF condition and those who started with the PLA condition. Independent-samples t-tests revealed no significant order effects for serve speed (t(11) = − 0.63, *p* = 0.539), spike speed (t(11) = − 0.73, *p* = 0.480), or speed-endurance (t(11) = − 0.74, *p* = 0.478), indicating that neither carryover nor learning effects influenced the supplementation outcomes.

## Discussion

This study examined the acute effects of low-dose caffeine supplementation (3 mg/kg) on serve speed, spike speed, and speed-endurance in elite male sitting volleyball players. The results showed that caffeine intake produced a moderate improvement in serve speed. Although this improvement did not reach statistical significance after correction for multiple comparisons. No significant effects were observed for spike speed or speed-endurance performance. These findings suggest that while low-dose caffeine may offer a potential ergogenic benefit for upper-body explosive movements such as serving, its effects on more complex or endurance-related actions appear limited in this specific athletic context.

In the current research, although the initial analysis indicated a statistically significant improvement in serve speed with caffeine ingestion (*p* = 0.028, d = 0.460), this effect did not remain significant after Bonferroni correction (adjusted *p* = 0.084). However, the medium effect size and directionally favorable confidence interval (95% CI: 0.21 to 3.02 km/h) suggest a trend toward significance, which warrants cautious interpretation and further investigation. Therefore, understanding how the complexity and nature of motor tasks influence the ergogenic effects of caffeine is crucial for interpreting performance outcomes. Our serve and spike results were inconsistent with prior reports. This discrepancy may be explained by differences in motor task complexity. This view is partly supported by the findings of a study on professional football players by de Almeida et al. [27] who reported that caffeine (5 mg/kg CAF) improved tactical decision-making performance in simple scenarios but had no or a negative effect in more complex time-pressured situations. In our context, the absence of improvements in spiking and endurance may similarly be linked to the fact that these tasks require not only muscular output but also rapid perceptual and decision-making processes. Since cognitive effects of caffeine such as improved vigilance or reaction time were not directly measured in this study, their potential moderating role remains speculative and represents an important direction for future research. Serving, by contrast, is a more controlled and repeatable task, whereas spiking involves real-time adjustments in timing, positioning, and trunk stability. These biomechanical and cognitive demands, compounded by the high prevalence of mechanical pain in sitting volleyball athletes, may limit the extent to which caffeine’s central and neuromuscular benefits can be translated into meaningful performance gains.

Another factor that may have influenced performance outcomes is the specific caffeine dosage and form used. The present study administered a moderate dose of 3 mg/kg of pure caffeine in powder form. A trend towards improvement in serve and spike performance was observed, although not statistically significant. This result aligns partially with findings from Nemati et al. [28], who reported improvements in serve and spike accuracy following caffeine intake, particularly at higher doses. Their dose–response analysis demonstrated that 6 mg/kg caffeine produced stronger effects compared to 3 mg/kg, especially in serving performance. Taken together, these results suggest that while a 3 mg/kg dose may yield improvements in serve performance, the effect may not be robust enough to survive statistical correction procedures possibly due to limited statistical power or individual variability in response. These findings suggest that higher caffeine doses may exert a more pronounced effect, particularly on skills requiring explosive power and precision.

The speed-endurance test applied in this study was designed to evaluate participants’ resistance to short-term, high-intensity efforts, serving as an indicator of anaerobic energy system efficiency [7]. In current study no significant difference was observed between caffeine and placebo use in the players’ speed endurance performances (*p* = 0.709, d = 0.111). To date, no study has directly investigated the effects of caffeine intake on speed-endurance performance in sitting volleyball or comparable Paralympic team sports. In literature, lack of data makes our findings particularly valuable and highlights a gap in the current literature. Additionally, post-speed-endurance test RPE values were 17.76 ± 1.58 in the L-CAF condition and 17.84 ± 1.51 in the PLA condition. The difference between conditions was not statistically significant (t= −0.113, *p* = 0.912). These values indicate that, regardless of caffeine ingestion, athletes perceived the exercise as very intense, which is consistent with the maximal effort nature of the protocol. In a recent study with women’s sitting volleyball players, Liang et al. [29] reported a significant positive correlation between RPE and average heart rate, supporting the validity of RPE as an internal load indicator in this population. Moreover, a recent narrative review emphasized that RPE-based workload monitoring is a critical tool for understanding performance demands and preventing injuries in overhead and wheelchair Para sports, including sitting volleyball [30]. These findings reinforce the importance of monitoring perceived exertion in Paralympic volleyball, both as a reflection of maximal effort and as a practical measure for training management.

Several previous studies involving Paralympic athletes have reported beneficial effects of caffeine on sprint performance [31, 32]. In particular, the study by Graham-Paulson et al. [32] observed that a 4 mg/kg dose of caffeine enhanced repeated-sprint performance in elite wheelchair rugby players. These results may serve as a valuable point of reference for sports such as sitting volleyball, which involve comparable movement demands. Marszalek et al. [7] highlighted the importance of anaerobic power in sitting volleyball players by reporting strong correlations with field-based sprint and speed-endurance tasks. However, the present findings suggest that low-dose caffeine (3 mg/kg) may not be sufficient to enhance performance in short-duration, high-intensity endurance efforts, which contradicts the positive effects frequently reported in the literature. One of the main reasons for this discrepancy may be that the study was conducted with elite athletes participating in sitting volleyball, a sport characterized by upper-limb-dominant movement. The study by Karayigit et al. [33] demonstrated that caffeine intake positively affected lower-body muscular endurance, whereas similarly strong effects were not observed for upper-body muscular endurance. Unlike sprint-based protocols, sitting volleyball relies almost exclusively on upper-limb propulsion, which may attenuate caffeine’s typical benefits observed in lower-limb-dominant sports.

Another possible reason why caffeine did not significantly improve speed-endurance performance may be that the nature of the exercise protocol did not allow its ergogenic effects to reveal themselves [34]. Proposed mechanisms include increased calcium release in muscle cells and CNS stimulation through adenosine antagonism [35, 36]. Caffeine may also elevate β-endorphins, reducing perceived exertion [37–39]. However, such effects are usually stronger in prolonged submaximal efforts than in short maximal tasks. The short duration and maximal intensity nature of the speed-endurance test used in this study may not have allowed sufficient time for these central effects of caffeine to influence performance. Lastly, the physiological adaptations of elite participants should also be considered. In these individuals who are accustomed to high training volume and intensity, the stress threshold of the central nervous system is higher. This may reduce or mask the effects of stimulants such as caffeine. These results indicate that the effects of caffeine on exercise performance are highly context-dependent and cannot be generalized through a single mechanism. Although caffeine can provide significant performance increases in some sports and certain types of exercises, it is not surprising that these effects are not seen in branches that require specific motor skills and upper body muscle activation, such as sitting volleyball. Since the test protocols applied especially to disabled athletes contain different conditions from the classical literature, the results should be interpreted with caution.

Sitting volleyball differs significantly from traditional team sports in terms of athletes’ body mechanics, postural strategies, and musculoskeletal loading, as movements are performed almost entirely with the upper extremities. To compensate for reduced lower-limb support, players develop specific adaptations to maintain trunk stability and mobility, which are often associated with increased muscle activation and a higher incidence of musculoskeletal pain [40, 41]. Although caffeine is known to reduce fatigue and perceived pain, its benefits may be limited when the pain originates from mechanical factors. In such cases, the neural benefits of caffeine may not be translated into improved performance. Furthermore, performance outcomes in sitting volleyball may vary according to individual anthropometric characteristics and disability profiles. For example, Cavedon et al. [42] reported that athletes with a greater hand span and longer unaffected lower limbs demonstrated superior upper-body strength and sprint performance. However, as no subgrouping based on disability classification was conducted in the present study, inter-individual variability could not be fully accounted for, which may have influenced the observed effects of caffeine.

This study has several limitations. (1) The small sample size restricts the generalizability of the findings; while power analysis suggested that 12 participants would be sufficient to detect large effect sizes, larger sample size are needed to reliably detect medium and small effects. (2) Only male athletes were included, limiting applicability to female populations. (3) Interindividual differences in caffeine response driven by genetic factors such as CYP1A2 polymorphisms and ADORA2A receptor sensitivity were not controlled for, which may further restrict the generalizability of the results. Finally, (4) the cognitive effects of caffeine intake (e.g., attention, arousal, decision-making) were not evaluated, despite their potential influence on performance outcomes. Despite these limitations, this is the first study to evaluate caffeine supplementation in elite sitting volleyball players.

Our findings highlight that task complexity and sport-specific motor patterns critically shape caffeine’s ergogenic effects. For Paralympic volleyball, these findings suggest that low-dose caffeine (3 mg/kg) may provide a small advantage in serving a skill where explosive upper-body power and repeatability are critical while being unlikely to improve more complex or endurance-related tasks. Coaches and practitioners should therefore be cautious when recommending low doses for performance enhancement and may need to consider higher doses or task-specific strategies, balanced against potential side effects and regulatory constraints. These insights extend existing knowledge by demonstrating that results from able-bodied or lower-limb dominant sports cannot be directly generalized to Paralympic contexts. Future research should recruit larger, sex-diverse samples, include genetic and employ different dose protocols. Studies incorporating cognitive measures such as attention and reaction time would also provide valuable insight into the mechanisms underlying performance outcomes in adaptive sports.

## Conclusions

In conclusion, this study provides the first evidence on the acute effects of low-dose caffeine supplementation in elite sitting volleyball players. While a 3 mg/kg dose of caffeine showed a trend toward improving serve speed, the effect did not remain statistically significant after correction for multiple comparisons, and no meaningful improvements were observed in spike or speed-endurance performance. These findings emphasize that caffeine’s ergogenic potential is highly context-dependent, shaped by task complexity, sport-specific motor demands, and the predominance of upper-limb actions in sitting volleyball. Although caffeine has consistently been shown to benefit lower-limb dominant and able-bodied sports, the present results caution against directly generalizing these outcomes to Paralympic contexts.

## Data Availability

The data that support the findings of this study are available on request from the corresponding author. The data are not publicly available due to privacy or ethical restrictions.

## Abbreviations

L-CAF: Low-dose Caffeine
PLA: Placebo
mi/h: Miles per Hour
km/h: Kilometers per Hour
s: Second
RPE: Rating of Perceived Exertion
CNS: Central Nervous System
BW: Body Weight
ISO: International Organization for Standardization
SPSS: Statistical Package for the Social Sciences
